# Mechanically interlocked 3D multi-material micromachines

**DOI:** 10.1038/s41467-020-19725-6

**Published:** 2020-11-24

**Authors:** C. C. J. Alcântara, F. C. Landers, S. Kim, C. De Marco, D. Ahmed, B. J. Nelson, S. Pané

**Affiliations:** Institute of Robotics and Intelligent Systems, ETH Zurich, Zurich, Switzerland

**Keywords:** Actuators, Magnetic properties and materials, Molecular machines and motors, Synthesis and processing, Design, synthesis and processing

## Abstract

Metals and polymers are dissimilar materials in terms of their physicochemical properties, but complementary in terms of functionality. As a result, metal-organic structures can introduce a wealth of novel applications in small-scale robotics. However, current fabrication techniques are unable to process three-dimensional metallic and polymeric components. Here, we show that hybrid microstructures can be interlocked by combining 3D lithography, mold casting, and electrodeposition. Our method can be used to achieve complex multi-material microdevices with unprecedented resolution and topological complexity. We show that metallic components can be combined with structures made of different classes of polymers. Properties of both metals and polymers can be exploited in parallel, resulting in structures with high magnetic responsiveness, elevated drug loading capacity, on-demand shape transformation, and elastic behavior. We showcase the advantages of our approach by demonstrating new microrobotic locomotion modes and controlled agglomeration of swarms.

## Abstract (web summary)

Mechanically interlocking dissimilar materials, such as metals and polymers, is a challenging yet promising pathway for designing and fabricating complex systems on the small scale. Here, the authors report a novel interlocking fabrication scheme and showcase the fabrication of microrobots via 3D-lithography.

## Introduction

Interlocks are used in many mechanical structures to create mutual physical dependence between distinct components. This is very common in macroscopic machinery and has also been widely adopted by chemists to develop molecular machines. Mechanically interlocked molecules such as catenanes, rotaxanes, and pseudo-rotaxanes have been used in complex mechanical nanosystems, such as molecular shuttles, switches, and related devices^1–3^. These systems are capable of conveying cargo at the nanoscale, controlling chemical processes, functioning as biosensors, serving as robotic end-effectors, and even as components of nanofactories. While many tasks have been demonstrated, mechanically interlocked molecular systems are limited to the nano-scale, hindering their exploitation in larger systems, such as MEMS and robotics^4^. A recent approach under investigation consists of arranging assemblies of these molecular machines to enable tasks at larger scales^5^. Despite their potential applications, the methods used to fabricate mechanically interlocked nanodevices rely on intricate organic synthesis protocols^6^ limiting their integration with other materials such as metals. Novel manufacturing approaches that can overcome these limitations would provide a wealth of new metal-organic structures.

In the micro- and nanorobotic domain, there has been a wide variety of examples of machines capable of swimming in different fluid environments^7^, transporting and releasing cargo^8–10^, and triggering chemical reactions^11^. Many complex architectures have been demonstrated, ranging from helices^12^ to scaffold^13^ and Archimedean screw pumps^8^.

Fabrication techniques, such as soft-lithography microtransfer molding allowed for the fabrication of closed-loop and simple interlocked single-material structures^14^. However, the realization of small-scale swimmers built on interlocked components made of combinations of dissimilar materials, such as soft and hard modules, remains unexplored. Interlocking dissimilar materials such as metals, polymers, and other families of materials will open many avenues in several fields and technologies, where multi-material components with different functionalities are necessary. Particularly, in the field of biomedical small-scale robotics, where more sophisticated highly integrated machines are demanded, the proposed approach will allow for the fabrication of devices that can incorporate multiple components, such as smart drug reservoirs, steering and locomotion units, parts with contrast agents or cell storages. The interlocking can also facilitate the programmed disconnection between components by integrating smart materials that can degrade upon certain stimulus, without disabling the entire machine.

To better showcase the need for such a construct that can combine intrinsically distinct materials, we introduce the problem of integrating two independent 3D geometries, one being entirely composed of metal, while the other is composed of pure gelatin, prepared without the addition of any crosslinking agent, and possibly loaded with drug molecules. This particular combination highlights several obstacles, including (i) the non- existence of any other method rather than mold casting capable of shaping non-modified gelatin (i.e., without chemical modification or the addition of cross-linkers) into 3D structures at the microscale^15^; (ii) the impossibility of performing surface metallization (i.e., electroless plating or vacuum deposition) of gelatin without compromising its mechanical and chemical features, nor of loading it with a magnetic volume comparable to that of a fully metallic structure; and (iii) the limited amount of drugs that can be coated on the surface of a fully metallic structure without adding an additional coating. However, by interlocking these two materials, a biocompatible system with elevated drug loading capabilities and maximized soft-magnetic responsiveness can be engineered. Combining these two materials represents a fabrication challenge that, if solved, would also enable the use of several other combinations of materials, including shape-responsive hydrogels^16^, metal-organic frameworks^17^, and multiferroic polymer composites^18^.

Previous works reporting on the fabrication of metallic microstructures mechanically interlocked relied on a combination of microcontact printing on curved surfaces, followed by electrochemical deposition^19–21^. Despite its potential, this method has been limited to metallic parts, and not yet integrated with soft components. Origami is a powerful technique that can circumvent some of these limitations. It has been used to shape metallic devices^22^, micro- and nanoelectromechanical systems^23^, and robots^24^. Recently, micromachines with pre-programmable shape morphing capabilities^25^ and compatibility with CMOS processing^26^ were reported, which may help to solve practical aspects regarding the mass production and functionality of microrobots. However, there are strong constraints regarding the adhesion between different materials, but also concerning the relation between their mechanical properties and the resulting attainable 3D geometries. Furthermore, this technique is limited to systems relying on compliant joints.

A different manufacturing paradigm, which we see as a complement to origami-based methods, consists of the fabrication of multifunctional structures by sewing, braiding, knitting, or weaving different classes of materials^27^. By adjusting the pattern, distribution, and chemical composition of the mesh, different properties can be engineered. Unlike origami-based methods, it does not pose any constraints on materials integration. However, weaving below the micrometer scale has not been demonstrated. The method introduced in this work is inspired by the fabrication of functional fibers. By combining it with the capabilities of 3D lithography, it is possible to interlock 3D fully metallic and polymeric objects at the microscale. Indeed, Park et al.^28^ recently demonstrated that mechanically interlocking is a promising approach to overcome materials limitations and enabling shape transformation in electronic devices.

3D template-assisted deposition was demonstrated to be a suitable way of reliably fabricating fully metallic and multifunctional polymeric microstructures^7,20–31^. In the field of medical microrobotics, the use of fully Fe structures is the most promising biocompatible approach for applications requiring enhanced magnetic performance^7,10^. However, they are limited in terms of advanced functionalities, such as cargo delivery, shape transformation, and controlled degradation, which is more easily achievable using soft structures^11,17,32^. Nevertheless, using a similar fabrication strategy, a 3D-template-assisted casting method was recently developed in our group. We demonstrated that any water-soluble material could be shaped with almost any arbitrary shape and feature sizes in the 5-μm range^15^. These two accomplishments are complimentary in the field of medical microrobotics, particularly in the case of magnetic actuation. Incidentally, the processing of metals and polymers are incompatible at the microscale using commercially available purely additive manufacturing tools.

In this work, we show that the subtractive manufacturing step present in template-assisted methods can ensure processing compatibility between metal and polymer 3D molding at the microscale. Theoretically, numerous interlocking strategies and designs could be exploited. However, we observed that there are constraints regarding the set of attainable combinations of independent shapes. The elliptical shape of the laser voxel used for 3D exposure has two negative consequences. The first is that it requires larger distances between the two shapes to prevent overlapping of the exposure absorption profile, which would otherwise cause distinct vias to fuse. The second is that it is hard to write large continuous surfaces, such as spherical domes. The repetitive laser exposure around the same space would permanently crosslink the positive photoresist, thus making it impossible to chemically develop it. These limitations on geometrical tolerances and on the design-workspace are mostly dependent on the exposure technique and do not have any impact on the technique herein presented. We demonstrate a manufacturing strategy to produce microrobotic devices by interlocking soft and hard materials by means of 3D lithography and electrodeposition. We use a well-established commercial device (Nanoscribe Photonic Professional GT, Nanoscribe GmbH), which relies on TPA. Multiple independent 3D vias can be generated inside a positive photoresist layer. Previously, it was demonstrated that it is possible to use positive photoresists in an oil immersion mode^7^. Compared to previous works, the three main advantages of this writing mode are that it enables the use of opaque substrates and thick photoresist layers and that the photoresist/substrate interface can be accurately detected. Electrodeposition requires a continuous electrical path to the substrate. Therefore, this optical configuration permits to design vias for electroplating, while still leaving other cavities present in the mold available for polymer casting. In order to ensure CAD fidelity, the only constraint is that the vias of each geometry do not overlap. As a result, two independent geometries consisting of different materials can be obtained in a single photoresist deposition step. To showcase the utility of our approach, we fabricated multiple interlocked microstructures, and investigated their behavior under magnetic fields.

## Results

### Fabrication of interlocked structures

**Fig. 1:**
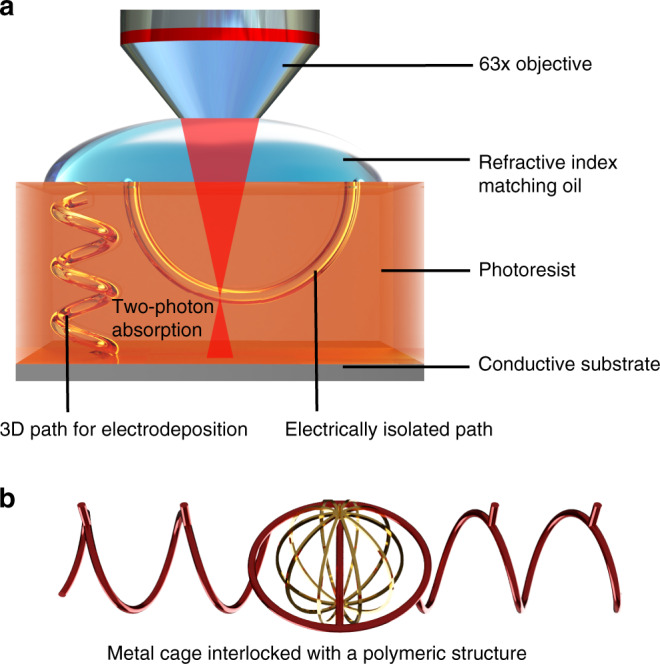
Fabrication of interlocked structures. **a** A polymeric bar is placed through the empty spaces of a metallic structure to interlock dissimilar materials. These two materials remain mechanically bound to each other through a closed ring. There is no design constraint over the shapes connected to the ring, as long as outlets are properly placed for material infusion through the microchannels. **b** To fabricate these structures, a high aperture objective is used in a two-photon absorption setup using a positive-tone photoresist immersed in a refractive index matching oil. This configuration allows for accurate detection of the photoresist/substrate interface. As a result, it is possible to design vias independently, which may be suitable for electrodeposition or not. The microchannels that are electrically isolated will remain unfilled after the electrodeposition step is concluded. As a result, a second casting step can be performed to mold water-soluble polymers. Consequently, two completely independent geometries composed of different materials can be obtained after dissolving the mold.

Figure 1a schematically illustrates the procedure for fabricating interlocked structures with dissimilar materials. Two different types of vias can be distinguished. The via that reaches the conductive substrate (helical channel in Fig. 1a) can be filled with a platable material (i.e., magnetic iron in this work) as it enables a direct contact between an electrolyte and the conductive substrate. The channel that is electrically isolated is used for mold-casting other materials such as polymers. If the channels are interlocked, the resulting metal and organic structures are also interlocked. Figure 1b shows a prototypical interlocked metal-organic structure consisting of a cage of an electrodeposited metal and a mold-casted complex polymeric architecture.

Here, we focused on two primitive shapes commonly used in microrobotics, metallic cages and helices, mechanically interlocked by a polymeric bar or a ring (Fig. 1a). By using this strategy, the fully metallic cage is free to rotate around it. However, when its rotation axis intercepts the bar’s plane, the structure is forced to tumble, resulting in motion, which is determined by the structure connected to the ring, but also to the number of bars, their shapes, and orientation. When the inner element does not intercept the bar connected to its respective ring, there would be little or no contribution towards net locomotion of the ensemble.

**Fig. 2:**
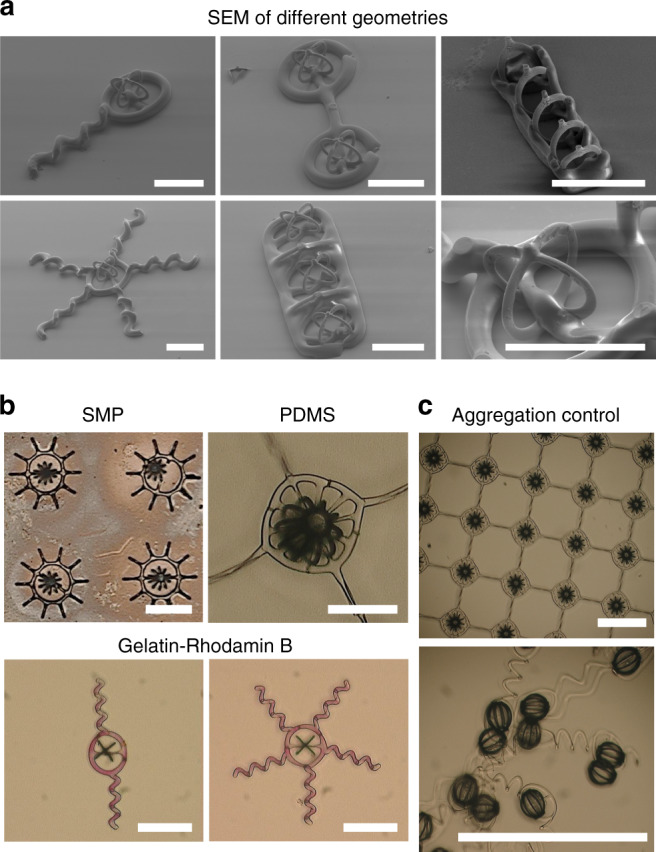
Fabricated mechanically interlocked metal-organic microstructures. **a** SEM images of 3D mechanically interlocked structures comprised of fully metallic Fe and PDMS components, highlighting the high-resolution features and possibility of designing completely independent shapes. The scale bar is 50 μm. **b** Optical images of structures comprised of different polymeric materials. The possibility of using different materials such as shape-memory polymers (SMPs), PDMS, and pure gelatin, possibly loaded with drugs, enables the fabrication of structures suitable for advanced functionalities, such as drug delivery and pre-programmable shape transformation. The use of an interlocking strategy guarantees process compatibility between metal and polymer deposition. As a result, the enhanced magnetic responsiveness and biocompatibility characteristic of fully metallic Fe structures can be harnessed in parallel. The scale bar is 80 μm. **c** In addition, hybrid microstructures can be mechanically stitched along the X-Y plane to prepare large meshes or filaments. This strategy is suitable to control the agglomeration of magnetic microparticles. The scale bar is 500 μm.

In Fig. 2a, scanning electron microscopy (SEM) images of different fabricated geometries are shown. These structures clearly illustrate that fully independent 3D shapes composed of different materials can be fabricated. In particular, the constraints (i.e., the path length of parallel filaments should match) associated with the electrodeposited part do not influence the geometry being generated by polymer casting. As shown in the same figure, the polymeric geometries can be designed independently of the magnetic element because they do not influence the electrodeposition step.

Despite the advantages of fully metallic iron structures, such as biocompatibility and enhanced magnetic performance, its use for advanced functionalities is hindered by their rigid nature. In Fig. 2b, we show that the capabilities of molding 3D microstructures fabricated using shape-memory polymers (SMPs), highly flexible silicon-based elastomers (i.e., PDMS), and pure gelatin (without crosslinkers), can be combined with the advantages of fully metallic structures. Unlike other techniques, our method does not pose major constraints on materials selection. For this reason, pure gelatin could be loaded with rhodamine-B, a model drug molecule, and interlocked with a fully metallic Fe cage without any modification in the process parameters.

**Fig. 3:**
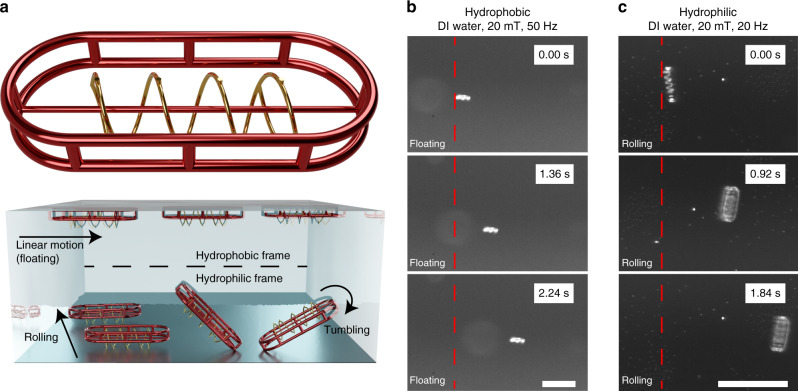
Mechanically interlocked micromachines with buoyancy control. **a** A polymeric frame placed around the long axis of a helical microswimmer can be used to allow metallic structures to fluid in low-density liquids such as DI water and blood. As represented in the schematic, the use of a hydrophobic frame (ecoflex) forces the structure to migrate towards the liquid/air interface, while a hydrophilic frame (PDMS) will cause it to sink and perform the rolling motion. To interlock the metallic and polymeric structures, the helical geometry is terminated by a half-segment with opposed chirality attached to both ends of the helix. By properly designing the pitch angle, an out-of-plane rotating magnetic field will result in a corkscrew motion^7^, thus resulting in forward motion. **b** When a hydrophobic frame (ecoflex) is used, hybrid microrobots can float and move forward when actuated using a rotating magnetic field. The round circle behind the structure is due to light reflection on the surface of the underlying substrate. **c** If a hydrophilic frame (PDMS) is used, the structure will sink towards the substrate and roll along its short axis when the same excitation is used. The scale bars are 150 μm.

An additional capability enabled by the approach introduced in this work is the possibility of controlling the aggregation of micro-sized particles, without affecting its overall mobility. Recent progress regarding the magnetic control of the collective behavior of small particles highlighted its prospects for biomedical applications^33–35^. Nevertheless, a recurring question related to the use of free particles is the reproducibility of the measurements and the risk of losing particles along the way, which is unavoidable, particularly when the particles are subjected to liquid flows. Concomitantly, it is important to highlight that physically restraining the motion of individual particles by embedding them in polymers, for example, possibly eliminate the advantages associated with the use of swarms of particles. For instance, the local vorticity generated by the collective motion of magnetic microparticles might help them to overcome liquid flows. In addition, the shape of particle swarms can readily adapt to overcome several obstacles, thus enabling them to move towards hard to reach locations, and readily reorganize to executive another task^34,35^.

In Fig. 2c, structures connected by highly flexible polymeric links are presented. This strategy does not constrain the individual motion of particles. Consequently, a few shortcomings associated with the use of swarms can be solved without compromising its advantages. In addition, the possibility of easily adjusting the initial configuration of the polymeric links and the density of magnetic elements may add a variable to control the collective behavior of magnetic microparticles. In applications where particle swarms are desirable, this strategy could prevent unwanted detachment and proliferation of particles to non-targeted areas. In parallel, the polymeric links can enable new strategies to execute specific tasks. For example, the tackiness of the polymer can be adjusted so that the swarm can attach to the walls.

### Mechanical interlocked micromachines with buoyancy control

A disadvantage of fully metallic microswimmers it that their weight results in an unavoidable interaction with solid substrates, thus inducing motion drift. Because our methods are compatible with a wide variety of polymers, we show that it is possible to combine metals with hydrophobic polymers. As a result, unwanted drift can be avoided by forcing the structure to float. In Fig. 3a, we introduce the possibility of altering the behavior of fully metallic structures in fluid through the placement of a polymeric frame around its long axis, akin to a rubber boat. We show that the use of a hydrophilic frame, such as PDMS, would not result in a change in buoyancy. Consequently, the behavior of a Fe/PDMS system in DI water would remain mostly unchanged, compared to its fully metallic counterpart. However, the use of hydrophobic polymers, such as ecoflex, a widely used material in soft robotics, would force the structure to move towards the water/air interface, thus to float.

Two different samples were prepared, one using an ecoflex (hydrophobic) frame and another using PDMS (hydrophilic). As discussed in other works^7,10^, fully metallic helices will sink and drift along a solid substrate when used in DI water, or liquids with similar viscosity. In Fig. 3b, it is demonstrated that the structure can effectively float and move forward. In this case, the parameters of the structure were chosen such that the easy magnetic axis of the magnetic part remains oriented towards the short axis of the geometry. Otherwise, the structure would try to tumble. Since it cannot overcome the liquid/air interface, the structure would not be able to propel itself forward. Instead, when a hydrophilic frame is used, the structure cannot perform any forward motion along its long axis. Instead, it can roll along its short axis or tumble along its long axis, depending on the configuration of the external magnetic field, as demonstrated in Fig. 3c.

### Multiple locomotion strategies of interlocked micromachines

**Fig. 4:**
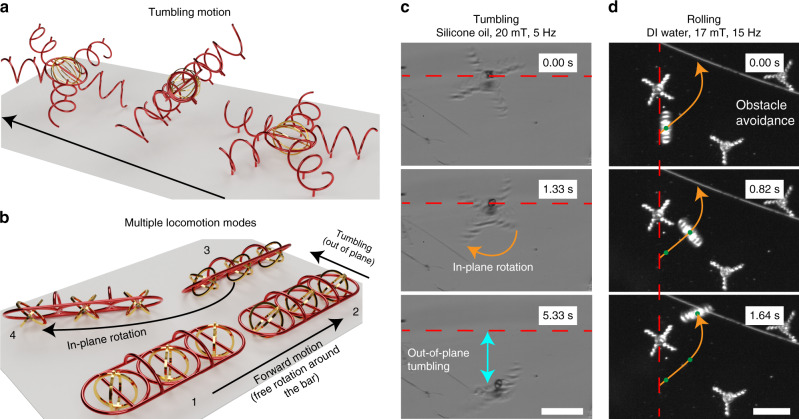
Multiple locomotion strategies of interlocked micromachines. **a** A cage-bar-ring structure connected to five helical legs can either rotate in-plane, which would not result in any net locomotion, or tumble out-of-plane. **b** A longer aspect ratio structure with multiple magnetic elements can either move forward, roll along its short axis, or change its direction by switching the direction of the external magnetic field, illustrating that multiple locomotion mechanisms can be exploited, despite the soft magnetic behavior of the metals used in this work. When compared to other rolling microrobots^36^, the method presented in this chapter introduces new possibilities and a considerably easier fabrication method. **c**–**d** Demonstration of an out-of-plane tumbling and rolling motion in silicone oil and DI water. The magnetic cage is free to rotate around the polymeric bar. If they intercept in-plane, the structure is allowed to rotate. If they intercept through the out-of-plane direction, the structure is forced to tumble, resulting in net locomotion (**c**). As schematized in (**b**), a rolling robot is capable of quickly changing its direction and avoiding obstacles using different locomotion modes (**d**). The scale bars are 150 μm.

The ability to switch through different locomotion strategies is another essential feature in microrobotics, where the same structure may be required to swim, roll, and tumble in order to perform tasks. In addition, the ability to reconfigure its shape in a passive or pre-programmed manner might be desirable when the same robot may need to move through narrow passages and restore its optimal shape^36^. In Fig. 4, we introduce the concept of using a cage (or helical) structure interlocked by bars to fabricate structures with predictable behaviors that can be easily switchable through an external magnetic field. In Fig. 4a, a cage-ring system connected to five helical legs is shown. Rotation of the metallic cage in directions that do not intercept its interlocking bar will not result in any net locomotion due to friction and because of the infrequent contact between the cage and the substrate. However, when the cage intercepts the bar, the overall system tumbles and moves forward as a result. Different patterns could be obtained by changing the relative angle between the bar and the orientation of the rest of the body. In-plane rotations are also possible, which serves to reorient the microstructure.

Conversely, the design in Fig. 4b shows that it is possible to overcome in-plane friction by reducing the number of non-magnetic contacts and increasing the number of actuation elements (i.e., cages). By properly adjusting the relative angle of the bar, tumbling around the short axis of the structure is favored in comparison to the rotation along its long axis. In terms of ease of fabrication, this strategy presents definitive advantages over other well-established technologies, such as the RodBots previously used in our group^37^. In addition, it presents an additional locomotion mode, which is the ability to perform forward motion along the long axis of the structure. Supplemental Videos 5 and 6 show the structures’ forward and tumbling locomotion at various frequencies. A frequency-dependent motion analysis, highlighting the reproducibility of the fabrication scheme, can be found in the supplemental information (Supplemental Fig. 4).

In Fig. 4c–d, a magnetic micromanipulation system was used to demonstrate that the motion scheme previously hypothesized is valid. In Fig. 4c, silicone oil was used for the sole purpose of producing better images and highlighting the contrast between the metallic and polymeric parts. As previously mentioned, when the cage intercepts the bar through in-plane rotation, the structure can be rotated, but without any resulting net locomotion. However, when the cage intercepts the bar through an out-of-plane rotation, the structure will tumble, thus moving forward. In Fig. 4d, we highlight the ability of rolling robots to effectively avoid obstacles by rolling forward and rotating around its out-of-plane axis.

## Discussion

The modular fabrication strategy presented in this work introduces the possibility of overcoming issues related to processing incompatibilities between different classes of materials, such as metals and hydrogels. We proved that by mechanically interlocking multi-material 3D, it is possible to embed advanced functionalities in microrobots without the need to compromise factors such as enhanced magnetic responsiveness, biocompatibility, and drug loading capabilities. Through these examples, we explored and demonstrated several advantages of mechanically interlocking multi-material objects. We also proved that this new fabrication paradigm could make it possible to fabricate systems that were never demonstrated with other techniques.

Compared to other techniques, such as four-dimensional direct laser writing^25^, self-folding structures^24^, and soft-lithography microtransfer molding^14^, the presented method imposes fewer constraints on attainable 3D shapes. Further, our approach is the only method that enables mechanical interlocking of metallic and polymeric (i.e., hard and soft) materials at this scale. The wide range of compatible materials makes the reported method a meaningful addition to the repertoire of 3D microfabrication techniques. Note that the two-photon lithography process is currently relatively slow, limiting mass production capabilities. However, recent advances, including the development of femtosecond projection systems, will likely make two-photon lithography a much faster process in the future^38^.

## Methods

### Materials

FeSO_4_.7H_2_O, FeCl_2_.4H_2_O, NH_4_Cl, gelatin from porcine skin, rhodamine B, glycerol, acetone, and isopropyl alcohol were purchased from Sigma-Aldrich. NOA63 (Norland Products Inc.) was bought from Thorlabs. AZ IPS-6050 and AZ 726MIF (Merck Performance Materials GmbH) were bought from MicroChemicals GmbH. Ecoflex 00-30 (Smooth-On, Inc.) and PDMS (Sylgard 184, Dow Corning) were provided by KauPo Plankenhorn e.K. and Suter Kunststoffe AG, respectively. Immersol 518f (Carl Zeiss Microscopy GmbH) was available from VWR International GmbH. All chemicals were used as received.

### Template fabrication

The templates were fabricated based on a recently reported protocol^7^. In brief, a commercial 3D nanolithography system (Nanoscribe Photonic Professional GT, Nanoscribe GmbH) was used to expose parts of a positive-tone photoresist. The latter is spin-coated on top of a 170-μm thick coverslip (3 cm in diameter), over which Ti (20 nm) and Cu (80 nm) were deposited by e-beam evaporation. The method presented in this work relies on the accurate detection of the photoresist/substrate interface, which is possible by contacting the objective (63x, Carl Zeiss AG) and the photoresist through a refractive-index matching oil (Immersol 518f, Carl Zeiss Microscopy GmbH). All the molds were prepared using either a 32 or 82-μm photoresist layer. AZ IPS-6050 was spin-coated at 2400 rpm for 18 s (32 μm) or at 1050 rpm for 13.5 s (82 μm) at a 300 rpm/s ramp. The samples were then placed on a hot plate at room temperature and heated up to 125 °C at a heating ratio of 15 °C per minute and baked at this temperature for 5 min (32 μm) or 15 min (82 μm), and then immediately transferred to a metallic surface at room temperature. When designing the CAD geometries, the filament thickness of the metallic, interlocking bar, and remaining polymer parts were respectively set to 0.5, 2, and 3, for 32-μm thick structures, or fixed at 2, 3, and 5 μm, for the 82 μm version. The minimum distance between metallic and polymeric parts was set to 10 μm to prevent overlapping of vias, and polymeric parts were distanced at least 4 μm from the substrate. The actual thickness and shape of the filaments are defined by the laser voxel, which is elliptical and varies as a function of laser power and exposure dose. Hatching and slicing of 3D CAD geometries were performed using the software DeScribe (Nanoscribe GmbH). The hatching/slicing distances were 0.3/0.3 μm (32 μm) or 0.3/1.0 μm (82 μm). All of these parameter values were empirically determined and should be re-tuned for different geometries. In either case, it is recommended to perform a laser power test when replicating these experiments in order to account for variabilities in the process. The positive-tone photoresist was then exposed using TPA from an infrared (780 nm) femtosecond laser at a scan speed of 2500 μm/s at a laser power set to either 32% (32 μm) or 50% (82 μm) of the maximum power that can be provided by the Photonic Professional GT. After exposure, the sample is thoroughly rinsed with DI water and blow-dried with nitrogen until immersol 518f is completely removed. The sample is then post-baked on a hot plate pre-heated at 100 °C for 140 s. Finally, the chemical development is performed by vertically dipping the sample in AZ 726MIF for 30 min (32 μm) or 90 min (82 μm) and then transferred to a container containing DI water. The sample is maintained therein until the metal electrodeposition step and never allowed to dry to prevent wetting issues, which could hinder electrolyte infiltration.

### Metal electrodeposition

The electrolyte used for iron electrodeposition was prepared by mixing FeSO_4_.7H_2_O (250 g/L), FeCl_2_.4H_2_O (42 g/L), and NH_4_Cl (20 g/L) at pH 2 for a total volume of 200 ml in a 400 ml beaker. A 2.5-cm magnetic stirring bar was used to continually stir the solution before and during electrodeposition at 100 rpm. A 5 cm × 5 cm platinum sheet was used as an inert counter-electrode and a double junction Ag/AgCl as a reference electrode. After the system is set, the substrate should be quickly transferred from DI water into the electrolyte, and immediately setting the potentiostat (PGSTAT204, Metrohm AG) to −0.95 V (vs. Ag/AgCl) to prevent substrate etching. The voltage was kept on until a current onset was observed, which was used as a cut-off deposition to stop the process and quickly transfer the sample to DI water, followed by thorough rinsing.

### Deposition of gelatin

Gelatin was prepared at a 10 wt.% in DI water. Stiffer structures can be prepared by increasing the weight percentage, while higher elongations can be achieved through the use of plasticizers, such as glycerol. Samples were successfully prepared by varying the concentration of gelatin up to 20 wt.% and by varying the glycerol content from 0 to 10 wt.%. The sample shown in Fig. 8c included 0.1 wt.% of rhodamine B to illustrate the possibility of using hybrid devices as a drug delivery tool. The sample was then immersed in the solution, which was kept above its gelling temperature while degassing the solution for ~5 min. The sample was then stored at ~4 °C for at least 2 h. Afterwards, gelatin was mechanically rubbed off the substrate

### Deposition of PDMS and ecoflex

The ratio between the polymeric base and curing agent used to prepare PDMS was set to either 5:1 (82 μm structures) or 10:1 (32 μm structures). Different ratios could be used to adjust the desired stiffness of the structures. Similarly, ecoflex 00-30 was prepared by mixing equal volumes of the two parts available in the kit. In both cases, the polymers were thoroughly mixed in a glass vial for up to 5 min and degassed until no bubbles were visible. The sample was then immersed in a shallow plastic container, on top of which roughly 5 ml of the polymer was poured. Polymer casting was then facilitated by vacuum infiltration. The sample was immersed in a vacuum chamber, which was consecutively pumped and vented until no bubbles were visible on top of the substrate, indicating that the polymer filled the microchannels. The substrate was then removed from the plastic container, and the excess polymer was removed using paper. The sample was then placed on top of the sulfonated paper to ease handling after the polymer hardens. PDMS was cured inside an oven at 65 °C for 4 h, and ecoflex was cured at room temperature overnight. Afterward, the thin film on top of the substrate was mechanically removed.

### Deposition of UV curable polymer

A few droplets of NOA63 were cast onto the photoresist template and left under vacuum for a couple of hours to allow NOA63 to infiltrate the template. A 1 mm-thick PDMS slab (Sylgard 184 Silicone Elastomer Kit), prepared by mixing the polymeric base and its curing agent in a ratio of 10:1 and cured at room temperature, was used to squeeze NOA63 between the AZ template and the PDMS slab. The conformal contact between the template surface and the PDMS slab was an essential step to eliminate any NOA63 residue from the AZ template’s surface. The template was then irradiated under UV light at 365 nm (2.7 mW cm^−2^) for 2 h at room temperature to cure NOA63. Afterward, the PDMS slab was gently removed. The mold was then removed by successfully washing the substrate with acetone, isopropyl alcohol, and water.

### SEM and EDX

A high-resolution field-emission SEM (Leo 1530, Carl Zeiss AG) was used for imaging and EDX mappings of iron/PDMS and iron/ecoflex structures. Prior to image acquisition, the samples were metalized with a thin layer of gold to avoid issues due to charge accumulation.

### Magnetic actuation

An 8-coil electromagnetic system arranged in a planar configuration (MFG- 100, Magnebotix AG) was used to generate magnetic field gradients and rotating magnetic fields. The measurements were performed by placing the samples in the center of the workspace of the magnetic field generator. The motion experiments were performed in a stacked Helmholtz coil setup at 2 mT (forward motion) and 4 mT (rotational motion) on a silicon wafer. The actuation frequency was increased until the structure lifted off the wafer surface.

## Supplementary information

Description of Additional Supplementary Files

Supplementary Information

Supplemental Movie 1

Supplemental Movie 2

Supplemental Movie 3

Supplemental Movie 4

Supplemental Movie 5

Supplemental Movie 6

## Data Availability

Raw data supporting the finding presented here are available upon request from the corresponding author.
