# Supplementary material for: Mechanically interlocked 3D multi-material micromachines
Source: Nat Commun. 2020 Nov 24;11:5957. doi: 10.1038/s41467-020-19725-6 (PMC7686494; doi:10.1038/s41467-020-19725-6)
Supplement: Supplementary file 1 — Description of Additional Supplementary Files [file 41467_2020_19725_MOESM1_ESM.pdf]

Title: Supplemental Video 1:

Description: Iron/PDMS microrobot demonstrating tumbling motion

Title: Supplemental Video 2:

Description: Iron/PDMS microrobot demonstrating rolling motion and obstacle avoidance

Title: Supplemental Video 3:

Description: Iron helix interlocked with an ecoflex (hydrophobic) frame

Title: Supplemental Video 4:

Description: Iron helix interlocked with a PDMS (hydrophilic) frame

Title: Supplemental Video 5:

Description: Frequency dependent forward motion of an iron/PDMS microrobot

Title: Supplemental Video 6:

Description: Frequency dependent tumbling motion of an iron/PDMS microrobot
