# Supplementary material for: Mechanically interlocked 3D multi-material micromachines
Source: Nat Commun. 2020 Nov 24;11:5957. doi: 10.1038/s41467-020-19725-6 (PMC7686494; doi:10.1038/s41467-020-19725-6)
Supplement: Supplementary file 2 — Supplementary Information [file 41467_2020_19725_MOESM2_ESM.docx]

**Supplemental Notes**

*C. C. J. Alcântara**^[[1]](#footnote-1)^*, F. C. Landers^1^, S. Kim, C. De Marco*, D. Ahmed, B. J. Nelson, and S. Pané**

**Manufacturing process** The manufacturing process can be better understood through the schematic in Supplemental Figure 1a. It shows a side view of microchannels embedded in a photoresist template. If a design similar to the ones presented in Figure 2a (with five helical legs connected to a ring) were to be designed, then thirteen outlets would be present. However, the number of holes leading to the substrate would remain unchanged despite the number of legs. Only along that section, metal would be electrodeposited. Supplemental Figure 1b shows a single hole on the conductive side, through which electrons can reach the substrate and ignite an electrodeposition process. The remaining holes on the non-conductive side remain free to be filled with other materials, such as polymers.

**Supplemental Figure 1: Schematics of Microchannels.** Schematic of microchannels embedded in a positive photoresist for the fabrication of hybrid microstructures. (a) Side view of a structure similar to the one shown in Figure 2a but connected to five helices. The cage is connected to the top and bottom bounds of the box, which represents the positive-tone photoresist layer. Note that there is no available path that connects the bar, ring, and helices to the top side of the bounding box. (b) If the top side of the box shown in (a) Would represent the conductive side of the substrate, a top view would show a single hole. Electrons would only be able to flow through paths connected to that outlet. In comparison, a bottom-view of the non-conductive side would show thirteen holes, all of which are available for polymer and electrolyte infusion. The pattern of these holes further exemplifies the selective character of the electrodeposition step and the importance of accurately identifying the photoresist/substrate interface.

The effectiveness of the method proposed in this work for the selective preparation of multi-material 3D structures can be confirmed through the energy dispersive X-ray (EDX) mappings shown in Supplemental Figure 2, where an Iron/PDMS hybrid system was used. It is expected that carbon is present in the metallic structure because of photoresist residues and contaminants during the electrodeposition process. Similarly, iron is highly prone to oxidation, and it is normal that its metallic form is electrodeposited in combination with hydroxides^7^. Nevertheless, the structures are highly pure with respect to other metallic and magnetic elements, such as Ni.

**Supplemental Figure 2: EDX analysis** EDX images of 3D mechanically interlocked structures, confirming that Fe is the only metallic element present in the cage, albeit carbon and oxygen contaminants. The comparison between the Si and Fe mappings confirms that the deposition of the metallic and polymeric parts is highly selective and that they are fully independent of each other.

**Agglomeration control** In Supplemental Figure 3, structures organized in a similar way to the ones in Figure 2c were used. In those images, a bright background was used to enhance the visibility of the magnetic elements. Due to the transparency of PDMS, the polymeric part of the structure can be hardly seen. When a rotating magnetic field at a low frequency of 0.1 Hz is used, each magnetic cage is free to move and rotate independently of each other (Supplemental Figure 3a). Under the excitation of a rotating magnetic field, the collective behavior of a particle swarm is highly dependent on the magnetic field’s input frequency. In Figure S3b, an ensemble of seven cages was subjected to a field of 15 mT rotating at 100 Hz. Under these conditions, it can be seen that parts of the structure tend to stretch and deform. Indeed, the particles are free to move, as opposed to other strategies which would constrain individual motion. However, because of the polymeric chain, the single element highlighted in the figure with a red arrow will remain part of the ensemble, despite its ability to move away from the central cluster.

**Supplemental Figure 3: Agglomeration control** Demonstration of stitched hybrid microstructures for agglomeration control. (a) Particles are free to move independently of each other. However, they remain agglomerated due to its polymer link (b).

**Locomotion of interlocked microrobots** Locomotion experiments of three different batches were performed to illustrate the reproducibility of the fabrication scheme. The structures were actuated at 2 mT for the forward motion and 4 mT for the tumbling motion in a stacked Helmholtz coil system on a silicon wafer. In the forward locomotion, the structures lifted off the substrate at 30 Hz, in the tumbling mode, the structures lifted off the substrate at 8 Hz.

**Supplemental Figure 4: Locomotion of Microrobots** Demonstration of reproducibility of the structures from three different batches through locomotion experiments. The structures were actuated in a stacked Helmholtz coil system (at 2 mT for the forward motion and 4 mT for the tumbling motion) at increasing frequencies until they lifted off the silicon wafer substrate. Each error bar represents tree measurements. (a) Tumbling locomotion between 0 Hz and 8 Hz (b) Froward locomotion between 0 Hz and 30 Hz. Each error bar represents the standard deviation of three trials.

1. Authors contributed equally to this work [↑](#footnote-ref-1)
